# Patient perspectives on pain care pathways: informing implementation with insights from trial evidence

**DOI:** 10.3389/fpubh.2026.1895073

**Published:** 2026-08-28

**Authors:** Lindsay A. Ballengee, Susan N. Hastings, Trevor A. Lentz, Katlin Harker, Steven Z. George

**Affiliations:** 1 Department of Orthopaedic Surgery, Duke University School of Medicine, Durham, NC, United States; 2 Duke Clinical Research Institute, Durham, NC, United States; 3 Center of Innovation to Accelerate Discovery and Practice Transformation (ADAPT), Durham Veterans Affairs Health Care System, Durham, NC, United States; 4 Department of Medicine, Duke University School of Medicine, Durham, NC, United States

**Keywords:** care pathways, implementation science, low back pain, patient preferences, qualitative research

## Abstract

**Background:**

Few studies have examined whether patients' perceptions of low back pain (LBP) care pathways are influenced by evidence of their effectiveness. To inform future implementation efforts, we conducted an exploratory qualitative study examining how individuals with low back pain interpreted two pain care pathways that emphasized non-drug treatment options: a physical therapy–focused model (Sequenced Care Pathway) and a navigator-led model (Pain Navigator Pathway). We explored how individuals with low back pain perceived the acceptability, feasibility, value, and fit of these approaches before and after reviewing comparative effectiveness trial results.

**Methods:**

Guided by the Theoretical Domains Framework, we conducted a qualitative study of individuals living with LBP using five small-group discussions and follow-up individual interviews with the same participants (*n* = 10). Participants were recruited from a patient and family advisory council at an academic medical center. Discussions explored perceptions of both pathways before and after participants reviewed trial results which indicated no pathway superiority for pain and functional outcomes.

**Findings:**

Participants viewed both pathways as acceptable; however, their interpretations and preferences were shaped primarily by prior experiences, access to care, perceived fit, and logistical considerations rather than trial results. Trial findings were typically interpreted as reinforcing existing preferences. The Pain Navigator Pathway was valued for coordination, support, and system navigation, while the Sequenced Care Pathway was viewed as structured and appropriate for specific patient contexts.

**Conclusions:**

In this exploratory qualitative study, participants' preferences were shaped by perceived fit, prior experiences, and healthcare system context, and remained largely stable after exposure to trial results showing no pathway superiority. These findings suggest that incorporating patient perspectives may help inform the design and implementation of pathway-based care, particularly in the commonly occurring instance when comparative effectiveness results do not identify a clearly superior approach.

## Introduction

Low back pain (LBP) remains one of the most prevalent and costly conditions worldwide and is a leading cause of disability (1). Clinical guidelines consistently recommend non-pharmacologic interventions such as physical therapy, exercise, and self-management approaches as first-line treatments. However, despite these recommendations, real-world low back pain care remains highly variable (2). Services considered low-value because of limited expected clinical benefit relative to potential harms, costs, or downstream utilization, such as routine imaging or spinal injections outside guideline-concordant indications, continue to be used at high frequencies, while access to recommended non-pharmacologic treatments is often delayed or limited (3, 4). These gaps highlight ongoing challenges in translating evidence into routine practice and ensuring that patients receive guideline-concordant care.

Care pathways are structured approaches to organizing and delivering care for a defined condition or patient population. In low back pain, pathway-based models have emerged as a strategy to improve delivery of evidence-based care by structuring treatment sequences, supporting coordination across services, and reducing variation in clinical practice (5). These pathways include sequenced approaches that prioritize non-pharmacologic interventions, as well as approaches that incorporate care navigation to guide patients through available treatment options. Many of these pathways have been evaluated using pragmatic and comparative effectiveness trials focused on outcomes such as pain, function, and healthcare utilization. Recent evidence from a 2025 Cochrane review suggests that pathway-based models provide modest improvements in pain and disability compared to usual care (6). However, most trials to date have compared pathway-based care to usual care which often lacks the coordinated and structured components characteristic of pathway-based models. As a result, there is limited evidence to guide decision-making when multiple pathway options are available.

When more than one reasonable care option is available, patient decision-making is often conceptualized as a process of integrating clinical evidence with individual values, preferences, prior experiences, and contextual constraints (7, 8). Shared decision-making and patient-centered care frameworks emphasize that evidence alone is rarely sufficient to determine the “best” option for an individual patient, particularly when available options have similar expected outcomes (7, 9). In this context, preferences are not fixed attributes but are formed through interpretation of information, assessment of perceived fit, and consideration of what is feasible within a patient's healthcare context (10). Qualitative methods are therefore well suited to examining how patients make sense of care pathway options and how comparative effectiveness evidence is interpreted alongside lived experience. Because the AIM-Back trial did not identify a clearly superior pathway, this study focused on how participants interpreted and formed preferences between two evidence-informed care options with similar expected outcomes, rather than on whether evidence of superior effectiveness changed decision-making.

Trials included in the 2025 Cochrane review provide important evidence about the effectiveness of pathway-based care compared to usual care and it is often assumed that patients will use this information to guide decision-making. However, two issues complicate this assumption. First, trials to date only provide information on care pathways vs. usual care and thus high-level evidence is lacking when patients are choosing between two different care pathways. Second, there is limited evidence on how patients interpret and respond to different available care models when trial evidence frequently does not identify one clearly superior option (6). Prior work suggests that patient decision-making is shaped not only by clinical evidence but also by prior experiences, expectations, perceived fit, trust, and contextual factors such as access to care and healthcare system complexity (7, 8). In practice, evidence may not function as a primary driver of behavior or patient preference. Instead, it may be interpreted alongside lived experience and used to confirm or question existing views.

To our knowledge, few studies have examined how patients interpret care pathways without knowing comparative effectiveness findings and how subsequent exposure to such evidence influences preferences for care. Understanding this process is important for informing implementation efforts, particularly in conditions like LBP where engagement and coordination across providers play a central role in whether evidence-based treatments are adopted in practice. Incorporating patient interpretations into pathway design and evaluation may help to identify features that support engagement and improve alignment between intervention design, patient values, and real-world needs.

Accordingly, the purpose of this study was to explore patient perspectives on two evidence-informed care pathways for low back pain that were implemented and tested for comparative effectiveness in the AIM-Back cluster randomized trial (11). AIM-Back included a physical therapy-focused Sequenced Care Pathway and a navigator-led Pain Navigator Pathway. In this study, we explored how individuals with lived experience of low back pain interpreted these two pathway options, how they formed preferences when presented with both pathways, and how exposure to trial results influenced their perceptions and decision-making. By examining patient perspectives before and after reviewing trial findings, this study provides exploratory insight into how patients make sense of pathway-based care when more than one evidence-informed option is available. These findings may inform future work to design and implement patient-centered, coordinated, and non-pharmacologic approaches to chronic pain care.

## Methods

### Trial overview

This exploratory qualitative study was conducted following the AIM-Back (Improving Veteran Access to Integrated Management of Back Pain) trial, a multisite, cluster-randomized pragmatic trial comparing two pathway-based models of care for low back pain across 17 primary care clinics within the Veterans Health Administration. The AIM-Back trial population was predominantly composed of Veterans with chronic LBP, including 90% meeting a frequency-based definition of chronic LBP and 60% meeting criteria for high-impact chronic LBP. Clinics were randomized to either the Pain Navigator Pathway (PNP), which used trained navigators to coordinate care and facilitate referrals to non-pharmacologic services, or the Sequenced Care Pathway (SCP), which emphasized structured, physical therapy-led care with telehealth-delivered components including physical activity training and psychologically informed interventions. Full details of the trial design, protocol, and pathway implementation have been reported elsewhere (5, 11, 12).

### Study design

We conducted an exploratory qualitative descriptive study using rapid qualitative analysis (RQA) guided by the Theoretical Domains Framework (TDF) (13). Participants reviewed both care pathways as part of an exploratory within-participant qualitative design intended to elicit interpretations of pathway features, perceived fit, and preference formation rather than to compare pathway effectiveness. This approach was selected to efficiently synthesize patient perspectives while maintaining analytic rigor and producing findings that are actionable for implementation (14, 15). The TDF provided a structured lens to examine behavioral, social, and environmental factors influencing patient perceptions of care pathways. This study followed best practices for qualitative research reporting and was reported using relevant items from the Consolidated Criteria for Reporting Qualitative Research (COREQ) checklist (16).

### Setting and participants

The study was reviewed and approved by the Duke University Institutional Review Board. All participants provided informed consent prior to participation. Participants were not enrolled in the AIM-Back trial and were recruited outside the Veterans Health Administration sites where the care pathways were implemented.

Participants were recruited from a Patient and Family Advisory Council affiliated with an academic medical center [e.g., DCRI Research Together (17)]. Council members were invited to participate through email outreach, and interested individuals contacted the study team or indicated interest through email. Of the 95 individuals approached, 14 expressed interest and 10 participated in the study. Because our IRB-approved protocol did not include collection of information from individuals who were invited but did not participate, we cannot determine whether participants differed from the broader group of advisory council members approached. This recruitment approach was selected to engage individuals with lived experiences of low back pain who could provide informed perspectives on care delivery and system navigation. Because the purpose of the study was exploratory rather than comparative, we sought participants who could reflect on pathway features, perceived fit, and practical considerations relevant to engaging in care for their low back pain.

Eligible participants were adults 18 years or older with a history of low back pain who were willing to discuss their perceptions of pain care pathways. Participants did not need to have received care through either AIM-Back pathway and were not required to be Veterans or patients within the Veterans Health Administration. Because participants were recruited outside the Veterans Health Administration, this study was not designed to characterize Veteran-specific preferences or draw conclusions about implementation within VHA settings specifically. Rather, one reason for convening this patient advisory group was to gather preliminary patient perspectives on whether care pathways developed and tested within VHA might be perceived as useful, acceptable, and feasible in non-VHA healthcare settings. More broadly, the study was intended to provide exploratory insight into how individuals with lived experience of low back pain interpret and form preferences between two evidence-informed care pathway options.

We conducted five small-group discussions with 10 participants total, followed by individual interviews with the same participants (*n* = 10). Because the same individuals participated in both data collection activities, small-group discussions and interviews were treated as complementary sources of within-participant data rather than independent samples. Small-group discussions were conducted before participants reviewed any AIM-Back trial results and were used to elicit shared perspectives and interactive responses to the pathway descriptions. Following completion of the focus groups, participants were presented with the AIM-Back trial results via PowerPoint slides and facilitated discussion and subsequently participated in individual interviews. These interviews explored how participants interpreted the trial findings and whether the results reinforced, refined, or changed their previously expressed views about each pathway.

Small group discussions lasted approximately 60 min each. Given the exploratory aims of the study and the focused nature of the research questions, sample adequacy was evaluated using thematic sufficiency rather than statistical representativeness. Thematic sufficiency was defined as the point at which iterative review of the transcript summaries and matrices did not identify new substantive themes related to pathway perceptions, perceived fit, or responses to trial evidence (18, 19). This determination was made through team discussion after review of the small-group discussion and interview summaries.

### Data collection

Data were collected using a semi-structured interview guide that was developed specifically for this study by the investigative team and informed by selected TDF domains relevant to patient engagement and care pathway decision-making. The guide included questions about initial reactions to each pathway, perceived benefits and concerns, anticipated barriers and facilitators to engagement, and reactions to trial results. The semi-structured discussion and interview guide is provided as Supplementary File 2.

Data collection occurred in two sequential phases. First, during small-group discussions, participants reviewed an overview of each care pathway and shared their initial perceptions before any AIM-Back trial results were presented. Second, after completion of the focus groups, participants reviewed comparative effectiveness trial results indicating no significant differences in pain and function outcomes between pathways via PowerPoint slides and facilitated discussion. Individual interviews then explored how this information influenced participants' perceptions and preferences.

Small-group discussions and individual interviews were facilitated by LB, who has training and experience in qualitative research, physical therapy, and implementation science. LB had no clinical relationship with participants. Participants were informed that the purpose of the study was to understand patient perspectives on low back pain care pathways and how trial evidence influenced their views.

All sessions were conducted virtually, audio-recorded, transcribed verbatim, and de-identified prior to analysis. Field notes were documented during or immediately after sessions to capture contextual observations and support interpretation during analysis.

### Data analysis

We applied a structured rapid qualitative analysis approach using summary templates and matrix-based data organization (14, 15). This approach is particularly useful in applied health services research where timely, actionable findings are needed.

Two analysts (LB, KH) independently reviewed each transcript and summarized key findings using a templated summary form aligned with TDF domains. The templates were informed by the TDF domains used to develop the discussion and interview guide, including beliefs about consequences, environmental context, social influences, and emotion, as well as study-specific questions about perceived pathway fit, anticipated barriers and facilitators to engagement, and responses to trial evidence. Summaries focused on identifying salient perceptions, decision-making factors, and responses to trial evidence. Completed summaries were then entered into a matrix using Microsoft Excel to facilitate within-case and cross-case comparisons across participants.

The analytic team conducted iterative reviews of the completed matrices to identify patterns within and across participants. Initial analytic observations were organized by TDF-informed summary domains and then compared across cases to identify recurring perceptions, points of divergence, and factors shaping pathway preference. Candidate themes were developed from these matrix summaries, reviewed against the underlying transcript summaries, and refined through team discussion and consensus. Theme development focused on both convergence and variation in participant perspectives, including how participants interpreted pathway features, perceived fit, and responded to trial evidence. The TDF was used as an initial organizing framework rather than a restrictive coding structure; final themes were developed inductively through review of the matrices and team discussion while remaining informed by TDF constructs relevant to patient engagement and decision-making.

Several strategies were used to enhance rigor and trustworthiness of the analysis, consistent with best practices for qualitative research (16, 20). Analyst triangulation was employed, with multiple team members involved in data summarization and interpretation to reduce individual bias and improve consistency.

An audit trail was maintained throughout the analytic process to document decisions related to coding, theme development, and data interpretation. Regular team meetings were used to discuss emerging findings, challenge assumptions, and refine interpretations. Formal member checking was not conducted. Instead, credibility and trustworthiness were supported through analyst triangulation, use of structured summary templates, matrix-based comparison across cases, maintenance of an audit trail, and regular team discussions to challenge assumptions and refine interpretations.

Reflexivity was an ongoing component of the analytic process. The multidisciplinary research team included individuals with expertise in physical therapy, pain management, and implementation science. Team members acknowledged that their clinical and research backgrounds could influence interpretation of the data, particularly related to preferences for non-pharmacologic care pathways. These perspectives were explicitly discussed during analysis to ensure that findings remained grounded in participant accounts rather than researcher assumptions (21).

## Results

Participants had a mean age of 57.1 years, were predominantly female (90%), and represented a range of racial and educational backgrounds (Table 1). Most reported experiencing low back pain within the past year, with 80% reporting current symptoms at the time of participation. Participants contributed data through both discussion groups and follow-up interviews; therefore, counts reflect unique participants (*n* = 10) rather than total interview encounters. Because this was an exploratory qualitative study and was not designed to estimate the prevalence of specific preferences, numerical counts are used sparingly and findings are interpreted as qualitative patterns rather than quantitative comparisons.

**Table 1:** Participant characteristics.

Characteristic	*n* (%) or Mean (SD)
Age, mean (SD)	57.1 (17.16)
Gender	
Female	9 (90%)
Male	1 (10%)
Race/Ethnicity	
White or Caucasian	7 (70%)
Black, African American, Hispanic	3 (30%)
Highest level of education	
Graduate degree	7 (70%)
College graduate	3 (30%)
Low back pain in past year	10 (100%)
Current low back pain	
Yes	8 (80%)
No	2 (20%)
Pain rating (0–10 scale), mean (SD)	5.30 (1.74)

Qualitative findings were organized into five primary themes describing how participants interpreted the care pathways, considered perceived fit, and formed preferences when presented with both care options. Themes were identified through iterative matrix-based analysis, with no new themes emerging in later transcripts, indicating thematic sufficiency. Table 2 provides high-level thematic summaries organized by TDF-informed analytic domains. Additional representative quotations supporting each thematic category are provided in Supplementary Table 1.

**Table 2:** Thematic summary by TDF-informed analytic category.

Domain	Pain Navigator Pathway (PNP)	Sequenced Care Pathway (SCP)
Perception of pathway	Viewed as supportive, flexible, and personal	Seen as clear and structured, but potentially rigid
Fit with patient needs	Aligned with holistic, individualized care values	Suitable for acute or first-time cases
Design and complexity	Appreciated for follow-ups and guidance	Praised for simplicity, but seen as too linear
Suggested changes	Desire for clearer navigator roles, more visuals	Desire for greater flexibility in sequencing
Emotional reactions	Described as hopeful and reassuring	Reassuring for some, but impersonal for others
Applicability	Broadly applicable, even with support networks	Better suited for straightforward or acute pain
Overall preference	Widely preferred due to adaptability and human element	Less preferred; good for certain situations

### Theme 1: preferences were shaped by prior experiences and remained stable after reviewing trial results

Participants consistently described their preferences for care pathways as being grounded in prior healthcare experiences, personal beliefs about treatment, and individual expectations of care. While trial results were viewed as informative and credible, they rarely altered preferences and were more often interpreted as reinforcing existing views. As one participant described:

“Knowing what the results are kind of matters to me… but you try to find what seems plausible and fits well with where you are in your life.”

Across participants, initial preferences often emerged during discussion of the pathway features and generally remained stable after participants reviewed the trial results. Rather than prompting reconsideration, trial findings were frequently described as confirming what participants already believed about the types of care that would work best for them. Many participants described the results as increasing their confidence in non-drug care options or validating beliefs they already held about avoiding medications or procedures. A smaller group expressed initial surprise that both pathways demonstrated improvement over time; however, these participants generally interpreted the evidence as consistent with an expectation that organized, non-drug care would be preferable to usual care.

Participants also interpreted the same neutral evidence differently depending on their prior experiences and perceived needs. Those with strong positive experiences with physical therapy often interpreted the findings as supporting continued confidence in a PT-focused approach. In contrast, participants who described fragmented care, difficulty coordinating services, or uncertainty about next steps tended to view the same findings as reinforcing the value of navigation and support. In this way, trial evidence appeared to be filtered through participants' existing beliefs, care histories, and sense of which pathway best fit their current situation.

### Theme 2: trial results aside, decision-making was driven by access, cost, and system-level constraints

Participants consistently described decision-making as driven by practical and contextual considerations, including access to care, cost, transportation, and healthcare system complexity. These factors were often described as more influential than pathway design or perceived effectiveness.

Across discussions and interviews, logistical barriers were described as overarching concerns regardless of pathway preference, including cost, scheduling, and insurance constraints. Participants who described prior negative experiences with fragmented care often framed these experiences as central to their current preferences. Financial burden was a prominent factor influencing engagement, with participants describing the cumulative and often unpredictable costs of care across visits and services:

“$1,400 a month… plus coinsurance… $2,000 ER visit…”

Participants also highlighted challenges navigating complex healthcare systems and obtaining timely care:

“It's very difficult to get quick access… it's a very complicated systems process.”

In addition, provider communication and clarity of next steps were described as critical drivers of decision-making. Participants described the cumulative burden of coordinating care across multiple providers, including scheduling delays, transportation challenges, and the need to repeatedly explain their medical history. These concerns shaped preferences by shifting attention from which pathway might be most effective in principle to which pathway felt most feasible, navigable, and sustainable in practice.

Overall, these contextual constraints were described as central determinants of engagement, often shaping what participants viewed as realistic or feasible care options. As a result, pathway feasibility was closely tied to real-world constraints rather than intervention design alone.

### Theme 3: the pain navigator pathway was valued for reducing system burden and supporting individualized care

Participants consistently described the Pain Navigator Pathway as addressing gaps in care related to coordination, communication, and system navigation. Many participants expressed a preference for features of the Pain Navigator Pathway, citing its personalization, emotional support, and ongoing guidance. Importantly, this preference was not generally framed as a belief that the pathway would produce superior clinical outcomes; rather, participants described the navigator role as reducing the work required to access and coordinate care.

Participants emphasized the importance of having guidance through the healthcare system:

“Having somebody that understands how the system works… and can guide you to that, I think, is a huge benefit.”

Participants also described the burden of navigating care independently and the need for someone to help coordinate across providers:

“I need someone… to have a 360-degree picture… not necessarily all these specialty people are on the same page…'

Some participants highlighted the importance of human connection, noting that early interaction and ongoing support would increase motivation and engagement in care. Participants also valued the pathway's flexibility and ability to connect them to multiple care options, allowing care to be tailored to individual needs and preferences.

In discussion groups, participants expressed strong enthusiasm for this model:

“How soon can we have this?”

Overall, participants found the Pain Navigator Pathway appealing not just for its components, but because it helped address gaps in coordination and continuity.

### Theme 4: the sequenced care pathway was viewed as structured and appropriate for specific patient contexts

Participants described the Sequenced Care Pathway as a structured and clinically grounded approach, particularly valuing its emphasis on physical therapy and stepwise care progression. A subset of participants were strongly attracted to certain aspects of the pathway, particularly its predictability and clarity.

Participants highlighted the value of a physical therapy–first approach and its focus on movement and rehabilitation:

“I would still choose the PT-first option… I'm such a believer in physical therapy…”

This emphasis on guided movement and education was viewed as an important component of recovery, particularly for individuals seeking a clear and proactive treatment approach.

However, rather than being universally preferred or rejected, the pathway was frequently discussed in terms of fit. Some participants noted that while the pathway may work well for others, it did not feel like a good fit for their own needs, particularly in the context of chronic or complex pain. This contrast highlighted that the same pathway feature could be interpreted differently depending on patient context. For participants seeking a clear rehabilitation plan, the structured nature of the Sequenced Care Pathway was appealing. For participants with longstanding or complex pain, however, the same structure could feel too narrow or insufficiently individualized.

Participants also suggested that the pathway may be particularly well-suited for individuals earlier in their care journey or those seeking a more structured, rehabilitation-focused approach. In contrast, participants with more complex or longstanding pain conditions often emphasized the need for flexibility and individualized care.

### Theme 5: trust in providers and healthcare system context influenced engagement

Across interviews and discussion groups, participants described trust in provider expertise and healthcare system context as important factors influencing engagement with care pathways. Trust was closely tied to prior experiences, with participants emphasizing the importance of trusted providers or encouragement from family members in shaping care decisions.

Participants also highlighted the importance of communication and feeling heard, noting that care models with follow-up or ongoing check-ins could increase their likelihood of engagement. In contrast, limited time with providers and fragmented care experiences were described as barriers to building trust.

Participants emphasized that perceptions of care quality could vary based on healthcare setting, geographic location, and provider availability:

“If you're in an academic center versus rural… that might make a difference.”

Trust was often described as influencing both expectations about care quality and willingness to follow through with recommended treatments. These findings highlight trust as a cross-cutting factor that shapes how patients interpret and engage with care pathways within the realities of their healthcare environment.

**Figure 1:**
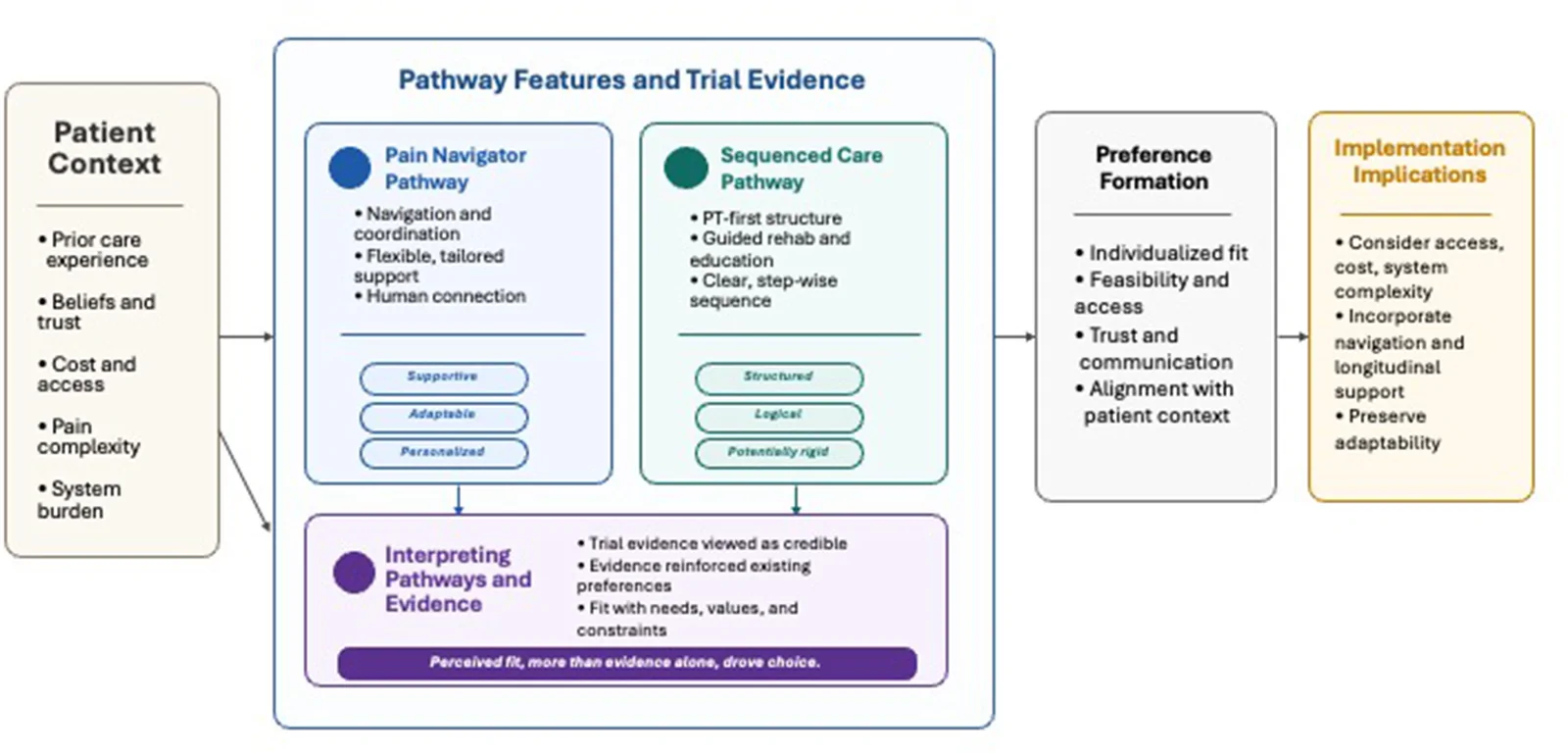
Conceptual model linking patient perceptions, pathway features, and implementation implications in the context of comparative effectiveness evidence showing no superior pathway.

Across themes, patient preferences were shaped by the interaction between prior experiences, perceived pathway features, practical constraints, and trust. Participants did not appear to interpret pathway information or trial evidence in isolation; instead, they evaluated both pathways in relation to what felt plausible, feasible, and aligned with their prior care experiences. Figure 1 illustrates how these patient perceptions map onto pathway features and implementation implications for future pathway-based care.

## Discussion

This qualitative study examined how patients interpret care pathways for low back pain and how exposure to trial results shapes preferences for care. Overall, preferences for care pathway selection were driven primarily by prior experiences and healthcare system context, with trial findings functioning more as validation than as a primary driver of decision-making. An important strength of this study is that initial pathway perceptions were elicited before participants reviewed the trial findings, allowing us to examine how comparative effectiveness evidence was interpreted relative to already established impressions rather than assuming preferences were formed only after exposure to the results. These findings provide an important complement to existing evidence on pathway-based care, which suggests modest improvements in outcomes compared to usual care but offers limited guidance when selecting between alternative pathway models. In the context of the AIM-Back trial, which did not demonstrate clear superiority between pathways, our findings suggest that patient-level factors such as prior experience, access, and perceived fit may be important considerations when designing and implementing pathway-based care.

While participants viewed the evidence from the AIM-Back trial as credible, trial findings rarely prompted reconsideration of existing preferences. Because the trial did not identify a clearly superior pathway, participants often interpreted the findings as supporting continued reliance on personal experiences, perceived fit, and existing beliefs about care. Thus, these findings should be interpreted as applying to a context of neutral comparative effectiveness evidence. Evidence demonstrating clear superiority of one pathway may have influenced preferences differently. Rather than shifting preferences, the results appeared to reinforce participants' comfort with the pathway they already favored. This pattern suggests that participants did not interpret trial evidence in isolation but instead evaluated it in relation to their prior experiences, expectations, and sense of which pathway best fit their needs. This aligns with prior work suggesting that patient decision-making is shaped not only by clinical evidence but also by experiential knowledge and personal beliefs about treatment (7, 8). These findings suggest that when comparative effectiveness results demonstrate similar outcomes across care models, patient preferences and contextual factors may play a particularly important role in shaping engagement, adherence, and pathway selection.

Participants also described decision-making as constrained by practical considerations such as cost, access, and healthcare system complexity. These findings are consistent with a growing body of literature demonstrating that healthcare decisions are shaped by structural and contextual factors that influence what care is realistically accessible (22, 23). In low back pain specifically, barriers such as limited access to non-pharmacologic care, cost, and care fragmentation have been identified as key drivers of variation in care and persistent use of services with limited expected clinical benefit when used routinely or outside guideline-concordant indications (1, 24–26). In this context, patient “preference” may be better understood as a function of both individual values and system-level constraints. Because these system-level constraints may differ across VHA and non-VHA healthcare settings, the specific barriers and facilitators identified by participants should be interpreted in relation to the healthcare context from which they were recruited. This interpretation is consistent with shared decision-making, patient-centered care, and constructed-preference perspectives, which emphasize that treatment decisions are shaped by the integration of evidence, values, preferences, relationships, and context rather than evidence alone (7–10).

Overall, participants evaluated each pathway in relation to their own needs and experiences. The Pain Navigator Pathway was valued for reducing the burden of navigating a fragmented healthcare system and for providing continuity and individualized support, whereas the Sequenced Care Pathway was seen as a structured, clinically grounded approach that may be better suited for certain patient populations, particularly those earlier in their care journey or those who prefer a more guided, rehabilitation-focused model. Together, these findings suggest that perceived pathway effectiveness may depend not only on clinical outcomes but also on alignment with patient context and perceived fit, highlighting the importance of flexibility in pathway design and the need to match care models to patient context (27, 28).

These exploratory qualitative findings suggest several considerations for future implementation of pathway-based care for low back pain. First, implementation strategies that rely solely on dissemination of evidence or clinical guidelines may be insufficient if contextual barriers such as access, cost, and system complexity are not addressed. This is particularly relevant in low back pain, where substantial gaps persist between guideline recommendations, real-world care delivery, and lack of a clearly superior treatment for LBP (2–4). This aligns with implementation science frameworks, such as CFIR and PRISM, which emphasize the role of environmental context and patient-level factors in shaping intervention uptake (27, 28).

Second, participants' favorable views of features associated with the Pain Navigator Pathway suggest that communication and longitudinal support may be important considerations in pathway design. Implementation efforts may benefit from incorporating strategies such as care navigation and patient-facing facilitation, which have been shown to improve engagement in complex care pathways and chronic disease management (29–31). These strategies may be particularly important in low back pain where patients often interact with multiple providers across care systems.

Third, the context-dependent perceptions of the Sequenced Care Pathway suggest the potential value of flexibility in pathway design and delivery. Rather than adopting a one-size-fits-all approach, health systems may consider offering multiple pathway options or allowing for adaptation within pathways to better align with patient needs. This approach is supported by evidence that patient decision aids improve knowledge, clarify values, and increase values-congruent choices when more than one reasonable option exists (9). In low back pain, stratified care models have also demonstrated improved outcomes and reduced costs by matching care to patient characteristics (32, 33). These findings support implementation approaches that preserve core pathway components while allowing flexibility in how patients are matched to or choose among care options.

This study has several limitations. First, the sample was small and had limited racial, ethnic, gender, educational, and Veteran status diversity. Although the sample was appropriate for the exploratory aims of the study and thematic sufficiency was reached, the small and relatively homogeneous sample may have limited the range of perspectives represented and may have prevented identification of additional themes. Second, participants were recruited from a patient and family advisory council at a single academic medical center, which may limit transferability to broader patient populations. In particular, advisory council members may have greater familiarity with research, or stronger interest in healthcare delivery than patients recruited from routine clinical settings. Although recruiting participants outside VHA was intentional given our interest in exploring how pathways developed and tested within VHA might be perceived in non-VHA settings, the findings should not be interpreted as representative of Veteran or VHA patient perspectives. This study focused on patient perceptions and preference formation and did not include a full assessment of non-VHA implementation considerations, such as reimbursement, staffing, workflow integration, organizational readiness, or local availability of services. Third, participants reviewed trial findings indicating no clear superiority between pathways; therefore, we cannot determine whether preferences would have remained as stable if one pathway had demonstrated substantially stronger effects. Fourth, the within-participant design was intentional and aligned with our aim of understanding how individuals form preferences when presented with more than one care option. However, exposure to both pathways may have introduced order effects, anchoring, or confirmation bias, and the findings should not be interpreted as a between-group comparison of pathway preference or effectiveness. Finally, participants were not enrolled in the original AIM-Back trial and were responding to descriptions of the pathways and trial results rather than direct experience receiving pathway-based care.

Finally, these findings have implications for how evidence is communicated and used in chronic pain management. Current approaches to chronic pain increasingly emphasize patient-centered, multimodal care that accounts for individual goals, preferences, and barriers to treatment. Rather than assuming that trial results will directly influence decision-making, our findings suggest that in the case of null or neutral trial findings evidence is interpreted through the lens of prior experiences and perceived fit. In this context, implementation efforts may need to focus not only on presenting evidence but also on helping patients understand how it applies to their individual circumstances, consistent with models of shared decision-making and patient-centered communication (7, 8). When randomized clinical trials frequently demonstrate small or non-significant differences between pathway models, patient preferences may warrant greater attention as one factor shaping engagement and uptake (6).

## Conclusion

In this exploratory qualitative study, preferences for two low back pain care pathways were shaped primarily by prior experiences, perceived fit, and healthcare system context. After participants reviewed AIM-Back trial results showing no clear superiority between pathways, trial evidence appeared to reinforce rather than determine pathway preferences. These findings suggest that incorporating patient perspectives may be important when clinical differences between interventions are small or uncertain. Integrating patient perspectives with trial results may improve alignment between evidence-based recommendations and real-world chronic pain care.

## Data Availability

The raw data supporting the conclusions of this article will be made available by the authors, without undue reservation.

## References

[1] Foster NE Anema JR Cherkin D Chou R Cohen SP Gross DP . Prevention and treatment of low back pain: evidence, challenges, and promising directions. Lancet. (2018) 391:2368–83. doi: 10.1016/S0140-6736(18)30489-6 29573872

[2] Qaseem A Wilt TJ McLean RM Forciea MA Clinical Clinical Guidelines Committee of the American College of Physicians Denberg TD. Noninvasive treatments for acute, subacute, and chronic low back pain: a clinical practice guideline from the American College of Physicians. Ann Intern Med. (2017) 166:514–30. doi: 10.7326/M16-2367 28192789

[3] Mafi JN McCarthy EP Davis RB Landon BE. Worsening trends in the management and treatment of back pain. JAMA Intern Med. (2013) 173:1573–81. doi: 10.1001/jamainternmed.2013.8992 23896698 PMC4381435

[4] Fritz JM Childs JD Wainner RS Flynn TW. Primary care referral of patients with low back pain to physical therapy: impact on future health care utilization and costs. Spine. (2012) 37:2114–21. doi: 10.1097/BRS.0b013e31825d32f5 22614792

[5] France C Cook CE Coffman CJ Tumminello C Choate A George SZ . The implementation of a pain navigator program in the department of Veterans Affairs' (VA) health care systems: a cluster randomized pragmatic clinical trial. Pain Med. (2024) 25:S83–90. doi: 10.1093/pm/pnae074 39514873 PMC11548858

[6] Docking S Sridhar S Haas R Mao K Ramsay H Buchbinder R . Models of care for managing non-specific low back pain. Cochrane Database Syst Rev. (2025) 3:CD015083. doi: 10.1002/14651858.CD015083.pub2 40052535 PMC11887030

[7] Elwyn G Frosch D Thomson R Joseph-Williams N Lloyd A Kinnersley P . Shared decision making: a model for clinical practice. J Gen Intern Med. (2012) 27:1361–7. doi: 10.1007/s11606-012-2077-6 22618581 PMC3445676

[8] Epstein RM Street RL. The values and value of patient-centered care. Ann Fam Med. (2011) 9:100–3. doi: 10.1370/afm.1239 21403134 PMC3056855

[9] Stacey D Lewis KB Smith M Carley M Volk R Douglas EE . Decision aids for people facing health treatment or screening decisions. Cochrane Database Syst Rev. (2024) 1:CD001431. doi: 10.1002/14651858.CD001431.pub6 38284415 PMC10823577

[10] Johnson EJ Steffel M Goldstein DG. Making better decisions: from measuring to constructing preferences. Health Psychol. (2005) 24:S17–22. doi: 10.1037/0278-6133.24.4.S17 16045413

[11] George SZ Coffman CJ North R Lentz TA France C Choate A . Sequenced care pathway vs pain navigator pathway for veterans with low back pain: the AIM-back cluster randomized clinical trial. JAMA Netw Open. (2026) 9:e264421. doi: 10.1001/jamanetworkopen.2026.4421 41926124 PMC13047465

[12] Lentz TA Coffman CJ Cope T Stearns Z Simon CB Choate A . If you build it, will they come? Patient and provider use of a novel hybrid telehealth care pathway for low back pain. Phys Ther. (2024) 104:pzad127. doi: 10.1093/ptj/pzad127 37756618 PMC10851867

[13] Atkins L Francis J Islam R O'Connor D Patey A Ivers N . A guide to using the theoretical domains framework of behaviour change to investigate implementation problems. Implement Sci. (2017) 12:77. doi: 10.1186/s13012-017-0605-9 28637486 PMC5480145

[14] Lewinski AA Crowley MJ Miller C Bosworth HB Jackson GL Steinhauser K . Applied rapid qualitative analysis to develop a contextually appropriate intervention and increase the likelihood of uptake. Med Care. (2021) 59:S242–51. doi: 10.1097/MLR.0000000000001553 33976073 PMC8132894

[15] Nevedal AL Reardon CM Opra Widerquist MA Jackson GL Cutrona SL White BS . Rapid versus traditional qualitative analysis using the Consolidated Framework for Implementation Research (CFIR). Implement Sci. (2021) 16:67. doi: 10.1186/s13012-021-01111-5 34215286 PMC8252308

[16] Tong A Sainsbury P Craig J. Consolidated criteria for reporting qualitative research (COREQ): a 32-item checklist for interviews and focus groups. Int J Qual Health Care. (2007) 19:349–57. doi: 10.1093/intqhc/mzm042 17872937

[17] Partner Engagement Duke Clinical Research Institute [Internet]. (2026). Available online at: https://dcri.org/solutions/partner-engagement (Accessed July 8, 2026).

[18] Guest G Bunce A Johnson L. How many interviews are enough?: an experiment with data saturation and variability. Field Methods. (2006) 18:59–82. doi: 10.1177/1525822X05279903

[19] Hennink MM Kaiser BN Marconi VC. Code saturation versus meaning saturation: how many interviews are enough? Qual Health Res. (2017) 27:591–608. doi: 10.1177/1049732316665344 27670770 PMC9359070

[20] Malterud K. Qualitative research: standards, challenges, and guidelines. Lancet. (2001) 358:483–8. doi: 10.1016/S0140-6736(01)05627-6 11513933

[21] Berger R. Now I see it, now I don't: researcher's position and reflexivity in qualitative research. Qual Res. (2015) 15:219–34. doi: 10.1177/1468794112468475

[22] Braveman P Egerter S Williams DR. The social determinants of health: coming of age. Annu Rev Public Health. (2011) 32:381–98. doi: 10.1146/annurev-publhealth-031210-101218 21091195

[23] Levesque JF Harris MF Russell G. Patient-centred access to health care: conceptualising access at the interface of health systems and populations. Int J Equity Health. (2013) 12:18. doi: 10.1186/1475-9276-12-18 23496984 PMC3610159

[24] Chou R Deyo R Friedly J Skelly A Hashimoto R Weimer M . Nonpharmacologic therapies for low back pain: a systematic review for an American College of Physicians Clinical Practice Guideline. Ann Intern Med. (2017) 166:493–505. doi: 10.7326/M16-2459 28192793

[25] Goertz CM George SZ. Insurer coverage of nonpharmacological treatments for low back pain-time for a change. JAMA Netw Open. (2018) 1:e183037. doi: 10.1001/jamanetworkopen.2018.3037 30646214

[26] George SZ Lentz TA Goertz CM. Back and neck pain: in support of routine delivery of non-pharmacologic treatments as a way to improve individual and population health. Transl Res. (2021). doi: 10.1016/j.trsl.2021.04.006 PMC8340679 33901699

[27] Glasgow RE Harden SM Gaglio B Rabin B Smith ML Porter GC . RE-AIM planning and evaluation framework: adapting to new science and practice with a 20-year review. Front Public Health. (2019) 7:64. doi: 10.3389/fpubh.2019.00064 30984733 PMC6450067

[28] Damschroder LJ Reardon CM Widerquist MAO Lowery J. The updated consolidated framework for implementation research based on user feedback. Implement Sci. (2022) 17:75. doi: 10.1186/s13012-022-01245-0 36309746 PMC9617234

[29] Freeman S Hirdes JP Stolee P Garcia J Smith TF Steel K . Care planning needs of palliative home care clients: development of the interRAI palliative care assessment clinical assessment protocols (CAPs). BMC Palliat Care. (2014) 13:58. doi: 10.1186/1472-684X-13-58 25550682 PMC4279598

[30] Chen M Wu VS Falk D Cheatham C Cullen J Hoehn R. Patient navigation in cancer treatment: a systematic review. Curr Oncol Rep. (2024) 26:504–37. doi: 10.1007/s11912-024-01514-9 38581470 PMC11063100

[31] Howitt L Jacob G Zucal G Smith J Crocker Ellacott R Sharkey S. Navigation support during transitions in care for persons with complex care needs: a systematic review. Healthcare. (2024) 12:1814. doi: 10.3390/healthcare12181814 39337156 PMC11431248

[32] Hill JC Whitehurst DG Lewis M Bryan S Dunn KM Foster NE . Comparison of stratified primary care management for low back pain with current best practice (STarT Back): a randomised controlled trial. Lancet. (2011) 378:1560–71. doi: 10.1016/S0140-6736(11)60937-9 21963002 PMC3208163

[33] Foster NE Mullis R Hill JC Lewis M Whitehurst DGT Doyle C . Effect of stratified care for low back pain in family practice (IMPaCT Back): a prospective population-based sequential comparison. Ann Fam Med. (2014) 12:102–11. doi: 10.1370/afm.1625 24615305 PMC3948756

