# Supplementary material for: Patient perspectives on pain care pathways: informing implementation with insights from trial evidence
Source: Front Public Health. 2026 Aug 28;14:1895073. doi: 10.3389/fpubh.2026.1895073 (PMC13562032; doi:10.3389/fpubh.2026.1895073)
Supplement: Supplementary file 1 [file Table_1.docx]

**Domain Map:** Factors that impact LBP care pathway choice.

**Study population:** People with the lived experience of LBP.

**Objective #1:** To describe the perceptions of PLE of LBP of two different care pathways for LBP care using domains from the Taxonomy of Patient Values and Preferences and the Innovation domain from CFIR.

| **Domain** | **Research Inquiry (i.e., what do you want to find out about this domain)** | **Sub-Domains** | **Sample Questions** |
| --- | --- | --- | --- |
| Perception of Sequenced Care Pathway | How do PLE perceive the SCP at first glance?  Positives of the SCP?  Negatives of the SCP?  How does the SCP fit within their view of treating LBP?  Additions to the SCP to make PLE want to use this pathway?  Deletions to the SCP to make PLE want to use this pathway?  What would make PLE excited to use the SCP?  What would make PLE nervous to use the SCP? | PLE perceptions of the design and complexity of the SCP.  PLE perceived likes and dislikes about the SCP.  PLE perceptions regarding how the SCP might be used in their specific situation.  PLE perceptions of important considerations for making the SCP more usable/feasible.  PLE overall emotions toward the SCP. | What comes to mind when thinking about the SCP?  What, if anything, do you like about the SCP?  What, if anything, do you not like about the SCP?  How, if at all, do you see using the SCP for your LBP?  What would your reaction be if this was the pathway you received when you got care for your LBP?  What, if anything, should be changed to make you want to use this pathway?  What, if anything, should be taken away to the SCP to make you want to use this pathway?  What, if anything, makes you excited/hopeful about using the SCP for your LBP?  What, if anything, would make you unsure or nervous about using the SCP for your LBP? |
| Perception of Pain Navigator Pathway | How do PLE perceive the PNP at first glance?  Positives of PNP?  Negatives of PNP?  How does the PNP fit within their view of treating LBP?  Additions to the PNP to make PLE want to use this pathway?  Deletions to the PNP to make PLE want to use this pathway?  What would make PLE excited to use the PNP?  What would make PLE nervous to use the PNP? | PLE perceptions of the design and complexity of the PNP.  PLE perceived likes and dislikes about the PNP.  PLE perceptions regarding how the PNP might be used in their specific situation.  PLE perceptions of important considerations for making the PNP more usable/feasible.  PLE overall emotions toward the PNP. | What comes to mind when thinking about the PNP?  What, if anything, do you like about the PNP?  What, if anything, do you not like about the PNP?  How, if at all, do you see using the PNP for your LBP?  What would your reaction be if this was the pathway you received when you got care for your LBP?  What, if anything, should be changed to make you want to use this pathway?  What, if anything, should be taken away from the PNP to make you want to use this pathway?  What, if anything, makes you excited/hopeful about using the PNP for your LBP?  What, if anything, would make you unsure or nervous about using the PNP for your LBP? |
| Preference for one pathway over the other | Which pathway would PLE choose if they were experiencing LBP? | PLE overall preference for pathway selection. | If you were seeking care for LBP and had a choice between two clinics – one that used the SCP and one that used the PNP, which clinic would you choose? (Follow up: Why didn’t you choose the other one?)  If not a clear preference - which factors would you want to know more about before making a choice? |

**Objective #2:**  To review results of AIM-Back trial describe acceptability/feasibility of the two different care pathways based on new evidence base.

| **Domain** | **Research Inquiry (i.e., what do you want to find out about this domain)** | **Sub-Domains** | **Sample Questions** |
| --- | --- | --- | --- |
| Perceptions of pathways considering new evidence regarding effectiveness | What do PLE think about the AIM-Back trial results?  How much difference, if at all, does it make knowing that the pathways have shown similar effectiveness? | PLE perceptions of useful and non-useful education about the trial results.  PLE about evidence-base, relative advantage, and source regarding each of the pathways. | How important is it for you to know the trial results before having a pathway preference?  What, if anything, do you wish you knew more about the trial results?  What has been most useful to you about learning the results?  What has been not so helpful in learning about these results? |
| Preference for one pathway over another | Which pathway would PLE choose if they were experiencing LBP now knowing trial results? | PLE perceptions of which pathway they might choose for their particular situation.  PLE perception of how their situation may or may not be helped by either of the pathways. | What, if anything, has changed about your perception of the pathways form the last time we talked about your preferences (i.e. in the first session)?  Which pathway would you now choose? Why? (Follow up: Why didn’t you choose the other one?)  What tends to be the most challenging when thinking about getting help for your LBP?  What factors, if any, might make it easier to use either pathway?  What are some difficult aspects of using either pathway that come to mind? |
| Acceptability of pathways | How suitable, satisfying, or attractive is the use of either the SCP or PNP among PLE of LBP? | How PLE describe their feelings about either pathway. | What reactions come to mind when you think about the SCP, PNP? |
| Intention to use pathways | To what extent, if at all, do PLE with LBP intend to use either of these pathways? | Intent to use pathways. | How much would the following factors be important for determining your preference (either pick 1-2 as most important and comment or briefly go through them all):   1. How long I had to wait to start care for my LBP 2. How far I had to travel to get my preferred pathway 3. Knowing that one pathway was more effective for my LBP symptoms 4. How much my copayment was |
