# Supplementary material for: Patient perspectives on pain care pathways: informing implementation with insights from trial evidence
Source: Front Public Health. 2026 Aug 28;14:1895073. doi: 10.3389/fpubh.2026.1895073 (PMC13562032; doi:10.3389/fpubh.2026.1895073)
Supplement: Supplementary file 2 [file Table_2.docx]

**Supplementary Table 1. Representative quotations by pathway and thematic category**

| **Thematic category** | **Pain Navigator Pathway (PNP)** | **Sequenced Care Pathway (SCP)** |
| --- | --- | --- |
| **Perception of pathway** | “The thing that stands out to me about the pain navigator pathway is that it’s focused on the pain... having someone dedicated to be checking in about that would be good... just being able to have one person help manage that would be helpful.”(PNP focus group participant) | “For me this one would work best... I would work better with the one on one with the physical therapist... watching how I exactly do it. For me personally, it would be best.” (SCP focus group participant) |
| **Fit with patient needs** | “It always appealed to me, the pain navigator pathway, because it would share decision making... Nobody would lose track of me. I would have one on one, someone to talk to.” (Individual interview participant) | “I think the sequence care pathway could be a good starting point. But... especially if you’ve already done physical therapy, that’s not that helpful.” (Individual interview participant) |
| **Design and complexity** | “I like that the pain navigator pathway has more options for treatment... having a real person who is the pain navigator telling you these are the options that you have.” (Individual interview participant) | “There’s just one route for the sequence care pathway... I think the sequence care pathway could be a good starting point.” (Individual interview participant) |
| **Suggested changes** | “Maybe if there’s like a pain navigator page on a website or something, there could also be resources on that page... here are the providers we recommend, or here’s like a search tool.” (PNP focus group participant) | “I would like to go to a physical therapist and have them do modalities on me first and then do physical therapy after... Make the physical therapy sessions longer.” (Individual interview participant) |
| **Emotional reactions** | “Many of us who have suffered from lower back pain... feel very isolated in complicated systems... [it may help to] talk about what is most emotionally helpful and be of some support to each other.”  (PNP focus group participant) | “There’s a psychological element to it when you have pain or chronic pain... it’s almost like you feel kind of alone. And that psychological part would be nice just to be like, you’re not crazy.” (SCP focus group participant) |
| **Applicability** | “If it was at the beginning of the journey instead of later... that’s when a pain navigator could be helpful.” (PNP focus group participant) | “I understand that the pain navigator and the sequence ones can be ideal for a person that broke a bone or pull a muscle. But people who [have] different comorbidities, the situation is a little bit more difficult to assess.” (Individual interview participant) |

Note: Quotations were selected from verbatim focus group and individual interview transcripts. Ellipses indicate omitted text. Speaker names and potentially identifying details have been removed.
